# Cost-effectiveness analysis of different rescue therapies in patients with lamivudine-resistant chronic hepatitis B in China

**DOI:** 10.1186/1472-6963-12-385

**Published:** 2012-11-08

**Authors:** Bin Wu, Jinfang Shen, Huafeng Cheng

**Affiliations:** 1 Department of Pharmacy Clinical Outcomes and Economics Group, Renji Hospital, affiliated with the School of Medicine, Shanghai Jiaotong University, Dongfang Road 1630, Shanghai, China

**Keywords:** Hepatitis B, Lamivudine resistance, Rescue therapy, Cost-effectiveness

## Abstract

**Background:**

Several rescue therapies have been used in patients with lamivudine (LAM)-resistant chronic hepatitis B (CHB); however, the economic outcome of these therapies is unclear. The object of the current analysis was to evaluate the lifetime cost-effectiveness of rescue therapies among patients with LAM-resistant CHB.

**Methods:**

A Markov model was developed to simulate the clinical course of patients with LAM-resistant CHB. From the perspective of Chinese health care, a lifetime cost-utility analysis was performedfor 4 rescue strategies: adefovir (ADV), entecavir (ETV) or tenofovir (TDF) monotherapy and combination therapy using LAM and ADV. A hypothetical cohort of 45-year-old patients with genotypic or clinical LAM-resistant CHB entered the model, and the beginning health state was LAM-resistant CHB without other complications. The transition probabilities, efficacy and resistance data for each rescue therapy as well as the costs and utility data were estimated from the literature. The discount rate (3%) utilized for costs and benefits. Sensitivity analyses were used to explore the impact of uncertainty on the results.

**Results:**

In LAM-resistant HBeAg-positive and HBeAg-negative CHB cohorts, TDF monotherapy and combination therapy were on the efficiency frontier for both positive and negative populations. Compared with no treatment, the use of combination therapy cost an additional $6,531.7 to gain 1 additional quality-adjusted life year (QALY) for HBeAg-positive patients and $4,571.7 to gain 1 additional QALY for HBeAg-negative patients. TDF monotherapy for HBeAg-positive patients, shows greater increase in QALYs but higher incremental cost-effectiveness ratio (ICER) in comparison with combination therapy. In probabilistic sensitivity analyses, combination therapy was the preferred option for health care systems with limited health resources, such as Chinese health care system.

**Conclusion:**

In Chinese patients with LAM-resistant CHB, combination therapy is a more cost-effective option than the competing rescue therapies.

## Background

The hepatitis B virus (HBV) infects about 350 to 400 million people worldwide (approximately 5% of the world’s population), including approximately 112 million Chinese people [1]. Chronic hepatitis B (CHB) infection has high morbidity and mortality because the ongoing viral replication increases the risk of cirrhosis and hepatocellular carcinoma [2]. Consequently, the suppression of HBV viral replication is one of the primary goals of antiviral therapy [3].

Several oral nucleoside antiviral agents, such as lamivudine (LAM), adefovir (ADV), telbivudine (TBV), entecavir (ETV) and tenofovir (TDF), have been approved for the treatment of CHB. Because of their superior efficacy and markedly low resistance, ETV and TDF have been recommended as first-line options for treatment of naïve CHB patients. [4] However, the high daily cost of ETV and TDF limits their widespread use in areas with limited health resources, such as in China [5]. LAM is still accepted as the main antiviral agent in these areas, largely because of its relatively low daily cost and well-established safety and efficacy profile [6]. However, long-term use of LAM is associated with a high rate of resistance (up to 70% by the end of 5 years) due to mutation at the YMDD motif [7]. Resistance to LAM could lead to a virological breakthrough and is associated with worse clinical outcomes [8]. Therefore, rescue strategies for dealing with LAM resistance are becoming an important issue in areas where LAM is widely used. The addition of ADV (a synthetic adenine nucleotide analog) or ADV monotherapy has demonstrated efficacy against LAM-resistant HBV in clinical trials and laboratory tests [9-13]. Although ADV monotherapy has lower daily costs than combination therapy, ADV monotherapy increases the risk of ADV resistance in patients with LAM-resistant CHB [14]. Because ETV and TDF have relatively high potency and high genetic barriers to resistance, switching to one of these two agents is an alternative [15-22]. However, because the rate of virological breakthrough is approximately 40% in LAM-resistant patients after 4 years of ETV therapy, [23] recent guidelines for managing CHB recommend that ETV be used as a strategy for dealing with LAM resistance only when the addition of ADV is not appropriate [3,24]. Although these rescue strategies have been introduced into clinical practice and have shown some health benefits for LAM-resistant CHB patients, many problems remain. The cost of these rescue strategies is a significant problem. To our knowledge, there are no report comparing the cost-effectiveness of different rescue strategies for patients with LAM-resistant CHB.

The objective of the current study was to evaluate the cost of 4 major rescue strategies for treating LAM-resistant CHB: ADV monotherapy, combination therapy using LAM and ADV, ETV 1mg monotherapy and TDF monotherapy. To compare the 4 rescue strategies over a lifetime, we developed a lifetime mathematical model for LAM-resistant CHB that incorporates clinical and economic data associated with CHB and its complications. The results from our study could be helpful for physicians and decision-makers.

## Methods

Using decision analytic and Markov modeling techniques, we constructed a mathematical model for HBeAg-positive and HBeAg-negative LAM-resistant CHB. We used this model to project the lifetime health and economic outcomes associated with CHB and its complications from the perspective of Chinese health care. The model was derived, in part, from our previous economic model for naïve CHB [5]. Cost and health outcomes were discounted at an annual rate of 3% [5]. The model was implemented using R software (version 2.12.2; R Development Core Team, Vienna, Austria).

Although multiple rescue strategies for LAM-resistant CHB have been evaluated in clinical trials, the most commonly recommended strategies are ADV monotherapy (10 mg/day), combination therapy using LAM (100 mg/day) and ADV (10 mg/day), ETV monotherapy (1 mg/day) and TDF monotherapy (300 mg/day) [6]. Therefore, these 4 rescue strategies were included to compare their cost and effectiveness over time. After patients develop LAM resistance, some will no longer receive antiviral therapy because of the high daily cost of the rescue agents. For this reason, we evaluated the outcome of no treatment as a common comparator for LAM-resistant CHB. Because the age of patients with LAM-resistant CHB varies widely, we evaluated the outcomes of patients with different ages at the onset of LAM resistance. Serum HBV DNA level is an ideal surrogate marker for antiviral therapies because of its independent predictive ability for CHB disease progression [25]. In this analysis, we use the serum HBV DNA level as the main endpoint for the various rescue strategies and for estimation of the risk of cirrhosis and hepatocellular carcinoma.

### Model overview

**Figure 1:**
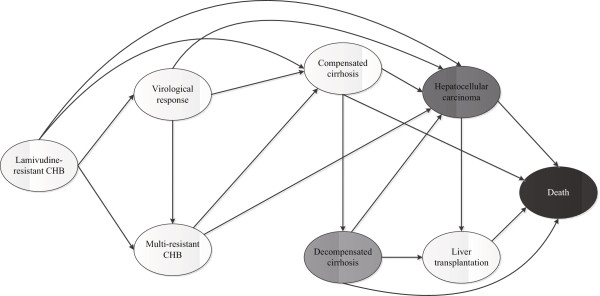
**Markov diagram of the LAM-resistant CHB disease model.** The ovals represent the eight mutually exclusive health states that the patient with LAM-resistant CHB might experience over a lifetime. Patients begin in the “lamivudine-resistant CHB” state and might transit between states or remain in their current health state during each 1-year cycle.

A Markov model consisting of 8 mutually exclusive health states was developed to track the potential lifetime courses of CHB. These states characterize the different health conditions associated with CHB, as shown schematically in Figure 1. A hypothetical cohort of 45-year-old patients with genotypic or clinical LAM-resistant CHB entered the model in the initial state, i.e., “lamivudine-resistant CHB.” Patients could move among eight health states after one year, as indicated by arrows in the Figure 1. According to the treatment response for each rescue therapy, defined as HBV DNA<300-400 copies/ml by quantitative PCR, a patient could transition from the initial state to either “virological response” or “multi-resistant CHB.” Patients with a “virological response” could also develop virological breakthrough due to new antiviral resistance and transition to the multi-resistant CHB health state. One important issue in the course of CHB is the progression from CHB to cirrhosis and hepatocellular carcinoma. The risk of disease progression is independently associated with the serum HBV DNA level [25]. Patients in any of the three states (“virological response,” “lamivudine-resistant CHB” and “multi-resistant CHB”) could develop compensated cirrhosis and hepatocellular carcinoma. However, patients in the virological response state would have a lower risk of disease progression than patients in the other two groups. Patients who develop compensated cirrhosis would be at risk for decompensated cirrhosis and hepatocellular carcinoma. Liver transplantation would be considered for patients in the decompensated cirrhosis and hepatocellular carcinoma states. The annual mortality of the progressed disease was also accounted for in the model for estimation of the health outcomes. The current analysis did not account for the natural mortality for other causes. We extrapolated the lifetime outcomes until all are dead or at the expected life-year (74 year of age in China) [26].

### Clinical data

The annual rates of progression were primarily derived from the literature, as shown in Table 1. The REVEAL-HBV study previously indicated that serum HBV DNA levels are an independent marker for predicting the progression of CHB (cirrhosis and HCC). Therefore, we assume that the progression depends on the serum HBV DNA level, regardless of nucleoside resistance. Patients with undetectable HBV DNA would be at the lowest risk for developing compensated cirrhosis and HCC. The proportions likely to receive liver transplantation for decompensated cirrhosis and HCC were derived from our previously reported article [5].

**Table 1:** Base-case annual probabilities associated with LAM-resistant CHB

**Initial state**	**Entered state**	**HBeAg (+)**		**HBeAg (−)**		**Source**
		**Estimate (%)**	**Range (%)**	**Estimate (%)**	**Range (%)**	
Lamivudine-resistant CHB	Compensated cirrhosis	2.7	1.6-3.8	6.2	2.8-9.7	[27]
	Hepatocellular carcinoma	0.4	0.3-0.6	0.4	0.3-0.6	[27]
Virological response	Compensated cirrhosis	0.5	0.3-0.8	1.2	0.7-2	[25]
	Hepatocellular carcinoma	0.2	0.1-0.4	0.2	0.1-0.4	[25]
Multi-resistant CHB	Compensated cirrhosis	2.7	1.6-3.8	6.2	2.8-9.7	[27]
	Hepatocellular carcinoma	0.4	0.3-0.6	0.4	0.3-0.6	[27]
Compensated cirrhosis	Decompensated cirrhosis	7.3	3.5-10	7.3	3.5-10	[28]
	Hepatocellular carcinoma	3.4	1-10	3.4	1-10	[28]
	Death	4.9	2-14	4.9	2-14	[28]
Decompensated cirrhosis	Hepatocellular carcinoma	3.4	1-10	3.4	1-10	[28]
	Liver transplantation	5	1-10	5	1-10	[5]
	Death	14.4	10-20	14.4	10-20	[5]
Hepatocellular carcinoma	Liver transplantation	5	1-10	5	1-10	[5]
	Death	23.3	20-30	23.3	20-30	[5]
Liver transplantation	Death	7	2-12	7	2-12	[5]

CHB, chronic hepatitis B.

Efficacy and resistance data (Table 2) for ADV monotherapy, combination therapy, ETV monotherapy and TDF monotherapy were estimated from clinical trials. A long-term clinical trial reported the four-year virological response and resistance of ADV monotherapy for HBeAg(+) and HBeAg(−) patients with lamivudine resistance [9]. Cumulative virological responses at four years were about 41.2% and 51.0% for the HBeAg(+) and HBeAg(−) cohorts, and the cumulative resistance rates were 51.6% and 42.4%, respectively. Data for combination therapy in patients with lamivudine-resistant HBeAg(+) CHB and data on resistance were derived from a clinical trial that included 132 lamivudine-resistant CHB patients; the cumulative virological response at two years was 68.0% [29]. For the HBeAg(−) cohort, the cumulative virological response at three years was 97.0% [30]. The cumulative resistance rate at four years was 8%; we assumed that there was no difference in resistance for the two cohorts. For ETV and TDF monotherapy, it was also assumed that the response and resistance rates for both cohorts was similar due to the absence of clinical data. The response rate for ETV in lamivudine-refractory CHB was estimated from a 96-week study [20]. The ETV resistance rate (21% at first year and 39.5% at second year) for patients with lamivudine resistance was estimated to be as high as 36.4%, based on a four-year assessment [23]. Because few studies have reported the efficacy of TDF for lamivudine-resistant CHB, we used data on TDF as a rescue therapy for patients following the failure of both lamivudine and adefovir. The cumulative virological responses at one and two years was 45.8% and 64.4, respectively [22]. The response to TDF was independent of mutations conferring ADV resistance. Due to no evidence of response and resistance data beyond the timeframe of clinical trial, we assume that no health benefit will be obtained from continuing the initial recue therapies beyond the clinical observation time, and patients who did not achieve a virological response would be considered resistant to the initial recue therapies and enter into the multi-resistant CHB state. We also assume that the resistances beyond the observation period were similar to those in the last year of the observation period. In our current analysis, we assumed the resistance rate was identical with relapsed rate.

**Table 2:** Base-case cumulative virological response and HBV resistance probabilities for each rescue therapy

**Rescue therapy**	**HBeAg (+)**		**BeAg (−)**		**Source**
	**Cumulative virological response (%)**	**Cumulative resistance (%)**	**Cumulative virological response (%)**	**Cumulative resistance (%)**	
ADV monotherapy	1st year: 20.4	1st year: 1.6	1st year: 33.3	1st year: 0	[9]
	2nd year: 33.2	2nd year: 20.7	2nd year: 44.0	2nd year: 16.9	[9]
	3rd year: 38.8	3rd year: 39.5	3rd year: 47.5	3rd year: 31.4	[9]
	4th year: 41.2	4th year: 51.6	4th year: 51.0	4th year: 42.4	[9]
ADV and LAM combination therapy	1st year: 47.0	1st year: 1.0	1st year: 60.0	1st year: 1.0	[29,30]
	2nd year: 68.0	2nd year: 1.0	2nd year: 76.0	2nd year: 1.0	[29,30]
		3rd year: 1.0	3rd year: 97.0	3rd year: 1.0	[29,30]
		4th year: 8.0		4th year: 8.0	[29]
ETV monotherapy^§^	1st year: 21.0	1st year: 1.0	1st year: 21.0	1st year: 1.0	[18,20,23]
	2nd year: 39.5	2nd year: 10.9	2nd year: 39.5	2nd year: 10.9	[18,20,23]
		3rd year: 25.2		3rd year: 25.2	[18,20,23]
		4th year: 36.4		4th year: 36.4	[18,20,23]
TDF monotherapy^§^	1st year: 45.8	1st year: 1.0	1st year: 45.8	1st year: 1.0	[22]
	2nd year: 64.4	2nd year: 2.0	2nd year: 64.4	2nd year: 2.0	[22]

§No separate data for HBeAg(+) and HBeAg(−) cohorts were reported; we assumed that the response and resistant data were similar for both cohorts.

### Cost and utility data

Direct health resource consumption was estimated from the perspective of Chinese health care, including the cost of medications, examinations, physician visits and laboratory tests (Table 3). Indirect costs such as lost productivity were not included. The annual costs of the four rescue therapies were calculated from the price of the antiviral drugs obtained from the Shanghai Municipal Bureau of Pricing. Several generic varieties of ADV are available on the current Chinese market. Because the median price of the generics was lower than Hepsera (the brand name for ADV from GlaxoSmithKline), we input the price of the generics into the model in the base-case analysis. At present, TDF is still awaiting approval from the Chinese State Food and Drug Administration, and no accurate price estimate is available. We assumed that the cost of TDF (300 mg dosage) would be similar to that of ETV ($6 per 500 mg dosage) in China because, as first-line antiviral options for naïve CHB, the costs of the two agents have been similar in other contexts [31,32]. Cost-related data for health states were obtained from previous studies [5,33]. All costs were converted to U.S. dollars (US $1 = CNY 6.5).

**Table 3:** Costs (2011 US$) and utilities input in the model

**Parameter**	**Base-case (range)**	**Source/comment**
Antiviral drug costs		
Lamivudine (100 mg)	2.07(1.86-2.27)	Local charge
Adefovir (10 mg)	2.31 (2.15-3.08)	Local charge
Entecavir (1000 mg)	10.20 (8–10.2)	Local charge
Tenofovir (300 mg)	6.00 (4.62-7.69)	The price and range were assumed
Annual health state costs		
Lamivudine-resistant CHB	138.7(104.62-209.23)	Assumed similar to naïve CHB [5]
Multi-resistant CHB	138.7(104.62-209.23)	Assumed similar to lamivudine-resistant CHB [5]
Virological response	138.7(104.62-209.23)	[5]
Compensated cirrhosis	235.37(209.23-313.85)	[5]
Decompensated cirrhosis	2122.55(1569.23-2615.38)	[5]
HCC	5914.78(5230.77-10461.54)	[5]
Liver transplantation	46152.94(44984.62-52307.69)	[5]
Post-liver-transplantation	7691.63(7323.08-9415.38)	[5]
Utilities (quality of life)		
Lamivudine-resistant CHB	0.52(0.5-0.7)	[34]
Multi-resistant CHB	0.52(0.5-0.7)	[34]
Virological response	0.71(0.65-0.8)	[34]
Compensated cirrhosis	0.57(0.5-0.7)	[34]
Decompensated cirrhosis	0.26(0.2-0.35)	[34]
HCC	0.31(0.24-0.38)	[34]
Liver transplantation	0.7(0.54-0.76)	[34]

CHB, chronic hepatitis B.

Health state utility values were derived from a recently published study using the standard gamble utility elicitation technique (Table 3) [34]. The utilities for lamivudine-resistant and multi-resistant CHB were assumed to be equal to those for naïve CHB.

The annual discount rate for costs and utilities was assigned as 3% in the current analysis [5].

### Analysis

To evaluate the uncertainty of parameter values and the robustness of the model, univariate sensitivity analyses were performed for each parameter in the model over the ranges shown in Tables 1, 2, 3. Ranges were sourced from reported literatures, such as 95 CI %, or a range±20% of the base case value when reported data were not available. Two-way sensitivity analysis was used to examine the uncertainty around the assumption associated with TDF, including the price and efficacy. To estimate the simultaneous impact of parameter uncertainty on the analysis, we also performed a probabilistic sensitivity analysis (PSA), in which distributions were assigned to the input parameters of the model (triangle distributions for costs, beta distributions for probability parameters and utilities). To identify the most cost-effective therapy over the range of threshold values, cost-effectiveness acceptability curves were plotted based on the result of the PSA. We generated a cost-effectiveness acceptability curve (CEAC) to identify the strategy with the greatest probability of it being cost-effective over a range of threshold values. CEAC could demonstrate the level of uncertainty associated with an option. The cost-effectiveness acceptability frontier (CEAF) is, however, constructed from the CEAC of the optimal option(s), and therefore only depicts uncertainty through the probability of not selecting the most cost-effective option [35]. Using scenario analyses, we evaluated the impact of age at initial rescue therapy by setting the age from 30 to 60 years.

## Results

The Markov model was used to estimate the clinical benefits in quality adjusted lifeyears (QALY) and the costs of the medication alternatives in the time horizon period. The comparison among the treatment alternatives was measured by the incremental cost-effectiveness ratio (ICER). The cost-effectiveness threshold developed is $11,034 (3× per capita GDP of China) and $38,376 (3× per capita GDP of Shanghai) for an additional quality-adjusted-life-year (QALY) gained according to WHO recommendation [36,37]. This recommendation has showed a potentiality of serving as a benchmark for threshold in this Asian context [38]. A discount rate of 3% per year was adopted for the costs and results.

### Base-case analysis

In the LAM-resistant HBeAg-positive cohort, treatment with TDF monotherapy resulted in 11.17 QALYs, and the lifetime cumulative incidence of compensated cirrhosis, decompensated cirrhosis, HCC and death was 30.7%, 11.7%, 13.1% and 25.5%, respectively. The other three competing rescue therapies yielded 9.25(ADV monotherapy), 10.58(combination therapy) and 9.43(ETV monotherapy) QALYs, respectively. In the LAM-resistant HBeAg-negative cohort, treatment with combination therapy achieved greater health benefits, which resulted in 10.48 QALYs followed by10.48 (combination therapy), 8.64 (ADV monotherapy)and8.51 (ETV monotherapy) QALYs, and a lifetime cumulative incidence of compensated cirrhosis, decompensated cirrhosis, HCC and death were 59.4%, 22.0%, 18.1% and 42.0%, respectively. The clinical and economic outcomes are presented in Table 4.

**Table 4:** Modeled clinical outcomes of life-time in lamivudine resistant CHB patient with rescue therapies

**HBeAg status**			**Positive**					**Negative**		
Treatment	No	ADV	ADV and LAM	ENT	TNV	No	ADV	ADV and LAM	ENT	TNV
Cumulative incidence of compensated cirrhosis^§^	0.51	0.49	0.39	0.47	0.31	0.80	0.76	0.59	0.76	0.53
Cumulative incidence of decompensated cirrhosis^§^	0.20	0.19	0.14	0.18	0.12	0.33	0.30	0.22	0.31	0.21
Cumulative incidence of HCC^§^	0.19	0.18	0.15	0.18	0.13	0.25	0.23	0.18	0.23	0.17
Cumulative mortality^§#^	0.41	0.38	0.30	0.37	0.25	0.64	0.58	0.42	0.59	0.41
Expected life-years	24.00	24.48	25.61	24.64	25.88	20.62	21.83	24.14	21.65	23.69
QALYs	8.77	9.25	10.58	9.43	11.17	7.76	8.64	10.48	8.51	10.36
Total cost ($)	9478.0	13011.9	21284.9	25387.0	27556.3	13097.2	16229.5	25552.8	27556.3	32270.9
Incremental cost ($)^&^	-	3533.8	11806.9	15908.9	22452.4	-	3132.3	12455.6	14459.2	19173.8
Incremental QALYs^&^	-	0.47	1.81	0.66	2.40	-	0.88	2.72	0.75	2.60
ICER^†^	-	7468.0	6531.7	24268.9	9359.8	-	3552.6	4571.7	19157.1	7370.4

§:Values are percentages, unless otherwise indicated.

#:Death is associated with hepatitis B.

&: “No treatment” was the baseline comparator.

†: ICER was calculated with the following equation: ICER=Incremental cost/Incremental QALYs. ICERs using the same 'no treatment' baseline.

**Figure 2:**
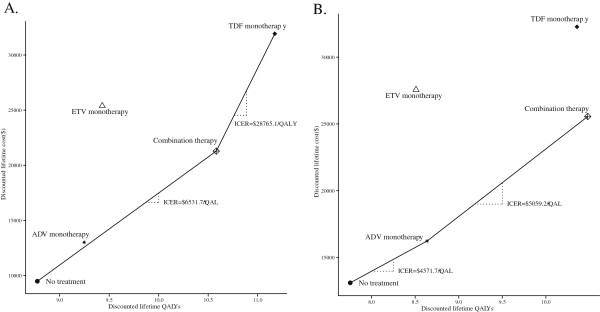
**Cost-effectiveness of rescue therapies for LAM-resistant HBeAg-positive (A) and HBeAg-negative (B) chronic hepatitis B.** The x-axis indicates the discounted lifetime quality-adjusted life-years (QALYs) for each therapy, and the y-axis indicates the total discounted lifetime costs (US dollar). The oblique line connects “no treatment” and the most cost-effective therapies; therapies above the straight lines were dominanated. Combination strategy uses LAM and ADV.

We evaluated the relative cost-effectiveness according to the QALYs and costs associated with the various therapies. The more expensive strategy was TDF in both cohorts. In the HBeAg-positive cohort, ADV and ETV monotherapy presented extended dominance and are not as effective (lower QALYs) and efficient (higher ICERs) than combination therapy. Combination therapy was also acceptable and had the lowest ICER of any of the rescue therapies. TDF monotherapy achieved extended success, with more QALYs provided than combination therapy but at a higher ICER. Among HBeAg-negative patients, TDF and ETV monotherapy were dominated; the combination therapy is more effective than ADV and presents an ICER below the threshold of China and Shanghai. The results are plotted in Figure 2.

### Uncertainty and scenario analyses

**Figure 3:**
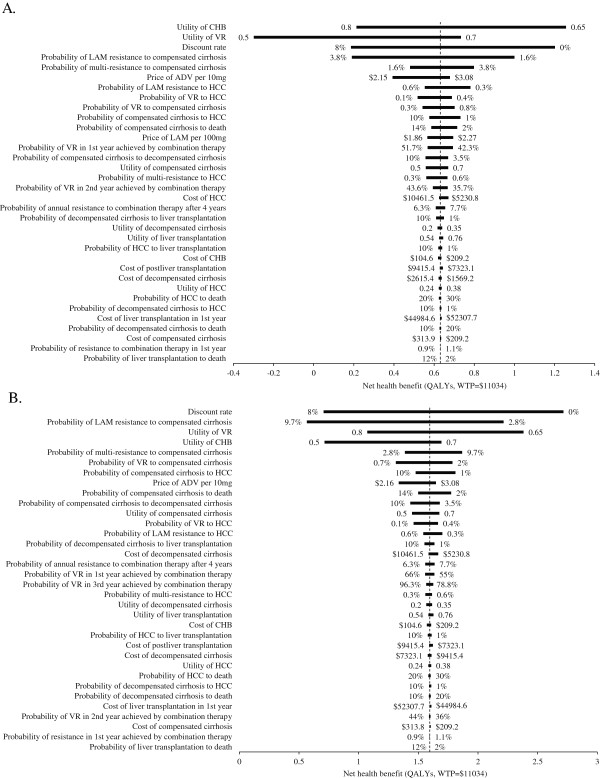
**Tornado diagram representing the net health benefit (QALYs, WTP=$11,034) determined by a one-way sensitivity analysis of combination therapy (ADV+LMV) vs. no treatment for LAM-resistant HBeAg-positive (A) and HBeAg-negative (B) chronic hepatitis B.** The vertical line represents the base-case value for the net health benefit under WTP=$11,034.

One-way sensitivity analyses revealed that the model was sensitive to some input parameters (Figure 3). In the two cohorts, the most substantial impact factors include the utilities of the CHB and virological response health states, the probability of LAM-resistant and multi-resistant CHB progression to compensated cirrhosis, and the price of ADV per 10 mg. Other parameters, such as cost of HCC and the probability of annual resistance to combination therapy beyond the four years, are less important.

**Figure 4:**
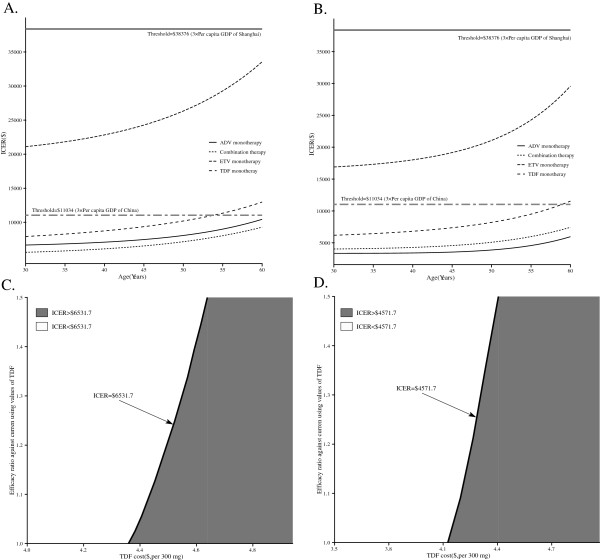
**Scenario and two-way sensitivity analysis.** The impact of age at initiation of LAM resistance on the incremental cost-effective ratio (ICER) of rescue therapies in comparison with “no treatment” for LAM-resistant HBeAg-positive **(A)** and HBeAg-negative **(B)** chronic hepatitis B: the x-axis indicates the age at initial rescue therapy, the y-axis indicates the ICER ($ per additional QALY), the bold horizontal two-dash and solid lines represent the thresholds for China and Shanghai City, respectively. Varying the TDF cost and efficacy leads to different incremental cost-effective ratio (ICER) of TDF monotherapy over “no treatment” for LAM-resistant HBeAg-positive **(C)** and HBeAg-negative **(D)** chronic hepatitis B: the x-axis indicates the different cost of TDF, the y-axis indicates the different efficacy of TDF (the ratio relatively to the current using values in the base-case analysis), the bold oblique lines represent that the ICERs for TDF strategy were equal to combination strategy, and grey and white areas indicates the ICERs for TDF strategy were higher and lower than combination strategy, respectively. Combination strategy uses LAM and ADV.

In a scenario analysis in which we evaluated the economic impact of age at the initiation of rescue therapy, the ICER of the four rescue therapies compared with no treatment increased with age (Figure 4A and B). For the HBeAg-positive cohort, the ICER of the combination therapy, which was dominant, fell below the threshold of $11,034 (3× per capita GDP of China) and $38,376 (3× per capita GDP of Shanghai), regardless of age. The TDF monotherapy was lower than the threshold of $38,376 but would be higher than $11,034 if the patient’s age was greater than 54. Among HBeAg negative, only TDF exceeds the thresholds of $11,034 but it is lower than the thresholds of $38,376.

Two-way sensitivity analysis for the variable TDF cost against efficacy is shown in Figure 4C and D. Comparing with “no treatment” for LAM-resistant HBeAg-positive (C) and HBeAg-negative (D) CHB cohort, TDF strategy must lower TDF price to $4.36 and $4.13 from an initial cost of $6.0 to yield a lower than $6531.7 and$4571 ICER (where the initial efficacy equals the base-case values), respectively. However, if the initial TDF price is $6.0, ICER of TDF strategy was always higher than $6531.7 and $4571 even when the efficacy was 1.5 times of the base-case values, respectively.

**Figure 5:**
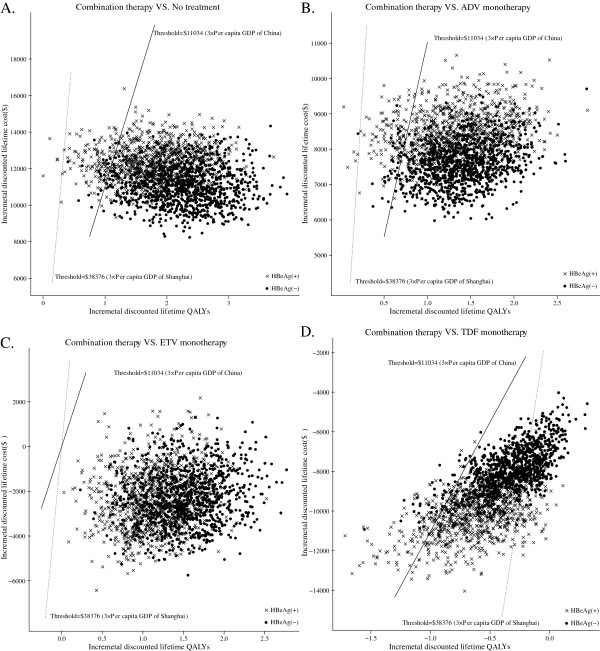
**Probabilistic results of the incremental cost-effectiveness differences between treatment with combination therapy (ADV+LMV) and with (A) no treatment, (B) ADV monotherapy, (C) ETV monotherapy and (D) TDF monotherapy for a cohort of 1,000 LAM-resistant HBeAg-positive and HBeAg-negative chronic hepatitis B patients.** The y-axis represents the incremental costs. The x-axis represents the incremental quality-adjusted life years (QALYs) gained. Dots that lie below the ICER threshold (the oblique lines) reflect simulations in which the cost per additional QALY gained with combination therapy was below the ICER threshold.

**Figure 6:**
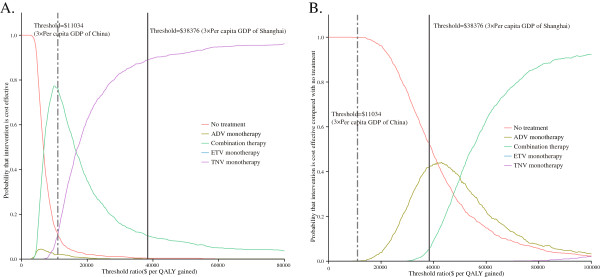
**Cost-effectiveness acceptability curve for rescue therapies for LAM-resistant HBeAg-positive (A) and HBeAg-negative (B) chronic hepatitis B.** The y-axis indicates the probability that a therapy is cost-effective across willingness to pay per QALY gained (x-axis). The bold vertical two-dash and solid lines represent the thresholds for China and Shanghai City, respectively. Combination strategy uses LAM and ADV.

A probabilistic sensitivity analysis of 1,000 simulations revealed the probabilities of meeting the ICER thresholds of $11,034 and $38,376 per additional QALY; the results are shown in Figure 5. For the HBeAg-positive cohort, the probabilities of cost-effectiveness being achieved by combination therapy compared with no treatment, ADV monotherapy, ETV monotherapy and TDF monotherapy were 87.6%, 89.7%, 100.0% and 11.2%, respectively, under the $11,034 threshold. The probabilities of cost-effectiveness under the $38,376 threshold were 99.7%, 99.5%, 100.0% and 89.5%, respectively. Among HBeAg-negative patients, the probabilities of cost-effectiveness being achieved with combination therapy were all over 95% in comparison with all other options except TDF monotherapy, regardless of whether the ICER threshold was $11,034 or $38,376. The CEACs showed that for the HBeAg-positive cohort the combination therapy and TDF monotherapy achieved the majority of probabilities of cost-effectiveness, and for the HBeAg-negative cohort no treatment yielded the majority of probabilities of cost-effectiveness when the WTP thresholds are $11,034 and $38,376 (Figure 6).

## Discussion

The high prevalence and morbidity of CHB pose heavy social and financial burdens for China. Because of limited health resources, most naïve CHB patients can afford only LAM as their first-line antiviral therapy; ETV and TDF are too expensive. Consequently, about 70% of patients will develop LAM resistance after 5 years. Selection of a cost-effective rescue therapy is an important issue not only for patients but also for physicians and decision-makers. The current economic analysis supplies health and economic information on the four main rescue therapies for LAM-resistant CHB patients.

The current analysis suggests that the initiation of rescue therapies for LAM-resistant CHB with combination therapy is likely to be more cost-effective than ADV, ETV or TDF monotherapy. But for patients HBeAg positive (Figure 5) the probabilistic sensitivity analysis comparing combination therapy with TDF shows that the probability to be costeffective is 11,2% for combination therapy compared to TDF under the $11,034 threshold . ADV monotherapy was the least expensive of the four rescue therapies, but its long-term health outcomes were poorest. Although ADV monotherapy was dominant, with the lowest ICER in HBeAg-negative patients, we believe that combination therapy is the optimal option. Results from PSA show it to be under the ICER threshold of $11,034; it would be more cost-effective compared to ADV monotherapy for about 96.1% of patients. In contrast, TDF monotherapy offered the greatest health outcomes for HBeAg-positive patients but was more resource-intensive than combination therapy (Table 4). In the HBeAg-positive CHB cohort, TDF monotherapy resulted in greater health benefits but was more expensive than combination therapy. Consequently, TDF monotherapy might be preferred in contexts with a high ICER or WTP threshold (such as $38,376 per QALY). However, among HBeAg-negative patients, TDF monotherapy did not show extended dominance; rather, it may contribute to a lower rate of virological response than combination therapy. Because few studies on TDF as a rescue therapy for HBeAg-negative CHB exist, the current estimation of the virological response rate was derived from a study in which only 33% of the patients had HBeAg-negative CHB. We suggest that TDF monotherapy could offer greater health benefits because the virological response was greater in the HBeAg-negative CHB cohort than in the HBeAg-positive CHB cohort [4]. These findings are generally consistent with the recommendations for managing LAM-resistant CHB, which involve the addition of ADV to LAM or TDF monotherapy [39-41].

Constrained economic conditions result in different treatment options. Each province and city in China has its own health care system, which determines medical coverage based on local economic conditions [42,43]. Our analysis shows how health decision-makers could select affordable, cost-effective therapies. For example, using 3× per capita GDP of Shanghai city as the WTP threshold [44], TDF monotherapy might be recommended for LAM-resistant HBeAg-positive CHB because this therapy achieves the greatest probability of cost-effectiveness in comparison with no treatment and other rescue therapies. However, from the perspective of the rest of China, when the threshold decreases to $11,034, combination therapy might be most affordable and cost-effective.

Our analysis includes two other important findings. First, based on recommendations by professional society guidelines and expert opinion, surveillance for resistance by monitoring of HBV DNA levels should be performed for all patients receiving nucleoside analogs. Our scenario analysis showed that an earlier age of rescue therapy initiation is related to a lower ICER for all four therapies. Second, our previous studies have shown that the utilities for CHB and virological response health states have substantial impact on the results of the model [5]. The current analysis finds a similar phenomenon. To the best of our knowledge, the prejudice and bias confronted by a person with CHB in China lead to worse sociocultural conditions than are faced by patients in western societies. CHB patients in China may be discriminated against in employment, marriage and education, as reflected in the lower utility values [45]. It would be reasonable to expect that improvement of the quality of life for patients with CHB would increase the QALYs.

The analysis presented here has several limitations that deserve consideration. First, most of the data for the virological response and resistance were not estimated from large-scale clinical trials. At present, ETV and TDF are widely used for naïve CHB patients; in the future it will be even more difficult to obtain large-scale data on the management of LAM-resistant CHB. Second, no clinical trial reporting virological response and resistance data lasted longer than five years. We used assumed data to calculate the lifetime outcomes. If long-term data become available, the accuracy of the results could be improved. Third, because no head-to-head trial has directly compared the efficacy of the four rescue therapies, our estimates were derived from various studies with varying design, patient cohort, follow-up, and quality. However, we examined the potential uncertainty of the variables with a sensitivity analysis. The final result was robust. Fourth, we did not evaluate potential treatment after a patient developed multiple resistance. In a constrained economic situation, such as China, many patients cannot afford expensive rescue therapies and would cease antiviral therapy after developing multiple resistance. Fifth, the current analysis did not include interferon-based strategies for dealing with LAM resistance. Some studies have suggested that a significant proportion of patients with LAM resistance who switched to pegIFNa-2a from LAM had a similar response to pegIFNa-2a as treatment-naïve patients [6]. Sixth, the utility scores for patients with LAM-resistant and multi-resistant CHB were assumed to be equal to those for naïve CHB. However, these scores might be worse in practice. One-way sensitivity analysis found that lower utilities of LAM-resistant and multi-resistant CHB would yield more health benefit. Finally, cumulative virological response and HBV resistance probabilities of ETV and TDF were assumed to be similar for both cohorts. If new data is available, an update analysis is necessary. However, the clinical data call for further study and a wider assessment. Nonetheless, because the current analysis reflects the current clinical practice for dealing with LAM-resistant CHB, we believe that the results will be helpful for patients, physicians and decision-makers.

## Conclusions

In conclusion, our analysis reveals that the use of combination therapy as a longer-term rescue therapy for LAM-resistant CHB is more cost-effective than ADV, ETV and TDF monotherapy in China. TDF monotherapy is a potential therapy under generous economic conditions. After development of LAM resistance, rescue therapy should be initiated as early as possible. Future research is needed to evaluate rescue therapies further, and economic analyses should be updated accordingly.

## Competing interests

Declaration of personal interests: All authors have nothing to declare. Declaration of funding interests: This work was supported by a grant from the Shanghai Pharmaceutical Association (no. 2010-YY-03), and a grant from Shanghai government (NO.08411951500), and a grant from School of Medecine, Shanghai Jiaotong University (NO.JYY0902).

## Authors’ contributions

Dr. BW and JS contributed to conception and design, Dr. HC contributed to analysis and interpretation of the data, Prof. JS contributed to collection and assembly of data. All authors read and approved the final manuscript.

## Pre-publication history

The pre-publication history for this paper can be accessed here:

http://www.biomedcentral.com/1472-6963/12/385/prepub

## References

[B1] Lee WM Hepatitis B virus infection N Engl J Med 1997 337 24 1733 1745 10.1056/NEJM199712113372406 9392700

[B2] Ganem D Prince AM Hepatitis B virus infection–natural history and clinical consequences N Engl J Med 2004 350 11 1118 1129 10.1056/NEJMra031087 15014185

[B3] Sorrell MF Belongia EA Costa J Gareen IF Grem JL Inadomi JM Kern ER McHugh JA Petersen GM Rein MF National Institutes of Health Consensus Development Conference Statement: management of hepatitis B Ann Intern Med 2009 150 2 104 110 19124811 10.7326/0003-4819-150-2-200901200-00100

[B4] Dienstag JL Benefits and risks of nucleoside analog therapy for hepatitis B Hepatology 2009 49 5 Suppl S112 S121 19399795 10.1002/hep.22920

[B5] Wu B Li T Chen H Shen J Cost-effectiveness of nucleoside analog therapy for hepatitis B in China: a Markov analysis Value Health 2010 13 5 592 600 10.1111/j.1524-4733.2010.00733.x 20561341

[B6] Marcellin P Sung J Piratvisuth T Avoiding and managing lamivudine resistance in chronic hepatitis B: current approaches and potential strategies including pegylated interferon Liver Int 2010 30 5 657 668 10.1111/j.1478-3231.2010.02207.x 20158610

[B7] Chang TT Lai CL Chien RN Guan R Lim SG Lee CM Ng KY Nicholls GJ Dent JC Leung NW Four years of lamivudine treatment in Chinese patients with chronic hepatitis B J Gastroenterol Hepatol 2004 19 11 1276 1282 10.1111/j.1440-1746.2004.03428.x 15482535

[B8] Ghany MG Doo EC Antiviral resistance and hepatitis B therapy Hepatology 2009 49 5 Suppl S174 S184 19399794 10.1002/hep.22900 PMC2707848

[B9] Lee JM Park JY Kim Do Y Nguyen T Hong SP Kim SO Chon CY Han KH Ahn SH Long-term adefovir dipivoxil monotherapy for up to 5 years in lamivudine-resistant chronic hepatitis B Antivir Ther 2010 15 2 235 241 10.3851/IMP1510 20386079

[B10] Chen EQ Wang LC Lei J Xu L Tang H Meta-analysis: adefovir dipivoxil in combination with lamivudine in patients with lamivudine-resistant hepatitis B virus Virol J 2009 6 163 10.1186/1743-422X-6-163 19818142 PMC2764700

[B11] Hezode C Chevaliez S Bouvier-Alias M Roudot-Thoraval F Brillet R Zafrani ES Dhumeaux D Pawlotsky JM Efficacy and safety of adefovir dipivoxil 20 mg daily in HBeAg-positive patients with lamivudine-resistant hepatitis B virus and a suboptimal virological response to adefovir dipivoxil 10 mg daily J Hepatol 2007 46 5 791 796 10.1016/j.jhep.2007.01.018 17321635

[B12] Yeon JE Yoo W Hong SP Chang YJ Yu SK Kim JH Seo YS Chung HJ Moon MS Kim SO Resistance to adefovir dipivoxil in lamivudine resistant chronic hepatitis B patients treated with adefovir dipivoxil Gut 2006 55 10 1488 1495 10.1136/gut.2005.077099 16461777 PMC1856440

[B13] Perrillo R Schiff E Yoshida E Statler A Hirsch K Wright T Gutfreund K Lamy P Murray A Adefovir dipivoxil for the treatment of lamivudine-resistant hepatitis B mutants Hepatology 2000 32 1 129 134 10.1053/jhep.2000.8626 10869300

[B14] Lee YS Suh DJ Lim YS Jung SW Kim KM Lee HC Chung YH Yoo W Kim SO Increased risk of adefovir resistance in patients with lamivudine-resistant chronic hepatitis B after 48 weeks of adefovir dipivoxil monotherapy Hepatology 2006 43 6 1385 1391 10.1002/hep.21189 16729316

[B15] Ryu HJ Lee JM Ahn SH Kim Do Y Lee MH Han KH Chon CY Park JY Efficacy of adefovir add-on lamivudine rescue therapy compared with switching to entecavir monotherapy in patients with lamivudine-resistant chronic hepatitis B J Med Virol 2010 82 11 1835 1842 10.1002/jmv.21898 20872709

[B16] Kim HJ Park JH Park DI Cho YK Sohn CI Jeon WK Kim BI Rescue therapy for lamivudine-resistant chronic hepatitis B: comparison between entecavir 1.0 mg monotherapy, adefovir monotherapy and adefovir add-on lamivudine combination therapy J Gastroenterol Hepatol 2010 25 8 1374 1380 10.1111/j.1440-1746.2010.06381.x 20659226

[B17] Lee JM Kim HJ Park JY Lee CK Kim Do Y Kim JK Lee HW Paik YH Lee KS Han KH Rescue monotherapy in lamivudine-resistant hepatitis B e antigen-positive chronic hepatitis B: adefovir versus entecavir Antivir Ther 2009 14 5 705 712 19704174

[B18] Karino Y Toyota J Kumada H Katano Y Izumi N Kobashi H Sata M Moriyama M Imazeki F Kage M Efficacy and resistance of entecavir following 3 years of treatment of Japanese patients with lamivudine-refractory chronic hepatitis B Hepatol Int 2010 4 1 414 422 10.1007/s12072-009-9162-x 20305760 PMC2836436

[B19] Goulis I Dalekos GN Entecavir monotherapy for lamivudine-refractory chronic hepatitis B Expert Rev Anti Infect Ther 2008 6 6 855 859 10.1586/14787210.6.6.855 19053898

[B20] Sherman M Yurdaydin C Simsek H Silva M Liaw YF Rustgi VK Sette H Tsai N Tenney DJ Vaughan J Entecavir therapy for lamivudine-refractory chronic hepatitis B: improved virologic, biochemical, and serology outcomes through 96 weeks Hepatology 2008 48 1 99 108 10.1002/hep.22323 18537189

[B21] Sherman M Yurdaydin C Sollano J Silva M Liaw YF Cianciara J Boron-Kaczmarska A Martin P Goodman Z Colonno R Entecavir for treatment of lamivudine-refractory, HBeAg-positive chronic hepatitis B Gastroenterology 2006 130 7 2039 2049 10.1053/j.gastro.2006.04.007 16762627

[B22] Patterson SJ George J Strasser SI Lee AU Sievert W Nicoll AJ Desmond PV Roberts SK Locarnini S Bowden S Tenofovir disoproxil fumarate rescue therapy following failure of both lamivudine and adefovir dipivoxil in chronic hepatitis B Gut 2011 60 2 247 254 10.1136/gut.2010.223206 21036792

[B23] Colonno RJ Rose RE Pokornowski K Baldick CJ Eggers B Yu D Cross A Tenney DJ Four year assessment of ETV resistance in nucleoside-naive and lamivudine refractory patients J Hepatol 2007 46 781

[B24] EASL Clinical Practice Guidelines Management of chronic hepatitis B J Hepatol 2009 50 2 227 242 10.1016/j.jhep.2008.10.001 19054588

[B25] Chen CJ Yang HI Iloeje UH Hepatitis B virus DNA levels and outcomes in chronic hepatitis B Hepatology 2009 49 5 Suppl S72 S84 19399801 10.1002/hep.22884

[B26] World Health Organization Life tables for WHO Member States 2011 http://www.who.int/whosis/database/life_tables/life_tables.cfm.

[B27] Fattovich G Bortolotti F Donato F Natural history of chronic hepatitis B: special emphasis on disease progression and prognostic factors J Hepatol 2008 48 2 335 352 10.1016/j.jhep.2007.11.011 18096267

[B28] Kanwal F Gralnek IM Martin P Dulai GS Farid M Spiegel BM Treatment alternatives for chronic hepatitis B virus infection: a cost-effectiveness analysis Ann Intern Med 2005 142 10 821 831 15897532 10.7326/0003-4819-142-10-200505170-00007

[B29] Yatsuji H Suzuki F Sezaki H Akuta N Suzuki Y Kawamura Y Hosaka T Kobayashi M Saitoh S Arase Y Low risk of adefovir resistance in lamivudine-resistant chronic hepatitis B patients treated with adefovir plus lamivudine combination therapy: two-year follow-up J Hepatol 2008 48 6 923 931 10.1016/j.jhep.2008.02.019 18433925

[B30] Manolakopoulos S Striki A Papatheodoridis GV Deutsch M Mela M Tzourmakliotis D Manesis EK Archimandritis AJ Effectiveness of long-term combination therapy with adefovir dipivoxil and lamivudine in patients with hbeag-negative chronic hepatitis b and lamivudine resistance Hepatology 2009 50 4 455

[B31] Veenstra DL Spackman DE Di Bisceglie A Kowdley KV Gish RG Evaluating anti-viral drug selection and treatment duration in HBeAg-negative chronic hepatitis B: a cost-effectiveness analysis Aliment Pharmacol Ther 2008 27 12 1240 1252 10.1111/j.1365-2036.2008.03691.x 18373637

[B32] Buti M Brosa M Casado MA Rueda M Esteban R Modeling the cost-effectiveness of different oral antiviral therapies in patients with chronic hepatitis B J Hepatol 2009 51 4 640 646 10.1016/j.jhep.2009.04.013 19576651

[B33] Guan ZQ Dong ZH Wang QH Cao DX Fang YY Liu HT Iloeje UH Cost of chronic hepatitis B infection in China J Clin Gastroenterol 2004 38 10 S175 S178 15602167 10.1097/00004836-200411003-00010

[B34] Levy AR Kowdley KV Iloeje U Tafesse E Mukherjee J Gish R Bzowej N Briggs AH The impact of chronic hepatitis B on quality of life: a multinational study of utilities from infected and uninfected persons Value Health 2008 11 3 527 538 10.1111/j.1524-4733.2007.00297.x 18179664

[B35] Barton GR Briggs AH Fenwick EA Optimal cost-effectiveness decisions: the role of the cost-effectiveness acceptability curve (CEAC), the cost-effectiveness acceptability frontier (CEAF), and the expected value of perfection information (EVPI) Value Health 2008 11 5 886 897 10.1111/j.1524-4733.2008.00358.x 18489513

[B36] Eichler HG Kong SX Gerth WC Mavros P Jonsson B Use of cost-effectiveness analysis in health-care resource allocation decision-making: how are cost-effectiveness thresholds expected to emerge? Value Health 2004 7 5 518 528 10.1111/j.1524-4733.2004.75003.x 15367247

[B37] Murray CJ Evans DB Acharya A Baltussen RM Development of WHO guidelines on generalized cost-effectiveness analysis Health Econ 2000 9 3 235 251 10.1002/(SICI)1099-1050(200004)9:3<235::AID-HEC502>3.0.CO;2-O 10790702

[B38] Zhao FL Yue M Yang H Wang T Wu JH Li SC Willingness to pay per quality-adjusted life year: is one threshold enough for decision-making?: results from a study in patients with chronic prostatitis Med Care 2011 49 3 267 272 10.1097/MLR.0b013e31820192cd 21224742

[B39] Pawlotsky JM EASL Clinical Practice Guidelines J Hepatol 2009 50 2 243 10.1016/j.jhep.2008.11.012 19081157

[B40] Sorrell MF Belongia EA Costa J Gareen IF Grem JL Inadomi JM Kern ER McHugh JA Petersen GM Rein MF National Institutes of Health consensus development conference statement: management of hepatitis B Hepatology 2009 49 5 Suppl S4 S12 19399804 10.1002/hep.22946

[B41] Liaw YF Leung N Kao JH Piratvisuth T Gane E Han KH Guan R Lau GK Locarnini S Asian-Pacific consensus statement on the management of chronic hepatitis B: a 2008 update Hepatol Int 2008 2 3 263 283 10.1007/s12072-008-9080-3 19669255 PMC2716890

[B42] Ling RE Liu F Lu XQ Wang W Emerging issues in public health: a perspective on China's healthcare system Public Health 2011 125 1 9 14 10.1016/j.puhe.2010.10.009 21168175

[B43] Li L The challenges of healthcare reforms in China Public Health 2011 125 1 6 8 10.1016/j.puhe.2010.10.010 21168176 PMC7118747

[B44] Cleemput I Neyt M Thiry N De Laet C Leys M Using threshold values for cost per quality-adjusted life-year gained in healthcare decisions Int J Technol Assess Health Care 2011 27 1 71 76 10.1017/S0266462310001194 21262069

[B45] Szabo SM Levy AR Davis C Holyoake TL Cortes J A multinational study of health state preference values associated with chronic myelogenous leukemia Value Health 2010 13 1 103 111 10.1111/j.1524-4733.2009.00573.x 19659707

